# Zinc Intake and Its Dietary Sources: Results of the 2007 Australian National Children’s Nutrition and Physical Activity Survey

**DOI:** 10.3390/nu4070611

**Published:** 2012-06-26

**Authors:** Anna M. Rangan, Samir Samman

**Affiliations:** Discipline of Nutrition and Metabolism, School of Molecular Bioscience, G08, The University of Sydney, Sydney, NSW 2006, Australia; Email: samir.samman@sydney.edu.au

**Keywords:** children, nutrition survey, zinc intake, food intake, Australia

## Abstract

The current Australian Nutrient Reference Values (NRV) use different Estimated Average Requirements (EAR) for zinc for adolescent boys and girls compared to the previous recommendations. The adequacy of zinc intakes of 2–16 years old children (*n* = 4834) was examined in the 2007 Australian National Children’s Nutrition and Physical Activity Survey. Zinc intakes were estimated from two 24-h recalls and compared with age- and gender-specific NRV. Food sources of zinc were assessed and compared with those of the 1995 National Nutrition Survey. The mean (SD) zinc intake was 10.2 (3.0) mg/day for all children. Nearly all children met the EAR for zinc except for 14–16 years old boys (29% did not meet EAR). Children (2–3 years) were at highest risk of excessive zinc intakes with 79% exceeding the Upper Level of Intake. Meat and poultry; milk products; and cereals and cereal products contributed 68% of total zinc intake. The contribution of cereals to total zinc intake has increased significantly since 1995, due to the greater market-availability of zinc-fortified breakfast cereals. We conclude that sub-groups of Australian children are at-risk of inadequate (boys 14–16 years) or excessive (children 2–3 years) zinc intakes, and monitoring of zinc status is required.

## 1. Introduction

Zinc is an essential trace mineral that has been identified as an at-risk nutrient in certain subgroups of the Australian population including toddlers and adolescents [1]. Very few studies have examined zinc intake and zinc status in Australia and many of these studies were based on small numbers of self-selected subjects and used methods that are now considered out-dated [1]. 

Zinc plays important roles in growth and development, immunity, neurological function and reproduction. Severe zinc deficiency was first observed in the 1960s [2], with reported symptoms that included growth retardation, hypogonadism and delayed sexual maturation. Other manifestations of zinc deficiency reported subsequently include high rates of infection (e.g., pneumonia and diarrhea) due to an immune deficiency, diverse forms of skin lesions, impaired wound healing, and night blindness [3,4]. Milder forms of zinc deficiency have been reported in developed countries such as Canada, where mild zinc deficient children were reported to have reduced growth velocity, reduced attention span and taste dysfunction [5].

Zinc is widely distributed in the Australian food supply. Based on 1995 data, meats, fish and poultry, and dairy foods are the major contributors to the diets of Australian children although cereals also contribute substantial amounts [6]. Since the voluntary fortification of cereals with zinc has become more common in Australia, it is important to determine how the increased availability of zinc in the food supply has affected total zinc intake.

Zinc requirements for the Australian and New Zealand populations were revised in 2005 as a set of Nutrient Reference Values (NRV) including a Recommended Dietary Intake (RDI), an Estimated Average Requirement (EAR) and Upper Level of Intake (UL) [7]. Compared to the previous recommendations, the revision resulted in an increase in average zinc requirements for adolescent boys and men but a decrease for adolescent girls and women. This was partly due to recognition that absorptive capacity for zinc varies across the genders and that men have significant losses in semen [7].

Prior to the 2005 revision of the NRV, zinc requirements were met by the vast majority of boys of all ages and girls under the age of 12. Approximately 40% of girls aged 12 to 18 years were at high risk of inadequate intakes as they did not meet 0.7 RDI (8.4 mg/day) in the 1995 National Nutrition Survey (1995 NNS) [8].

The 2007 Australian National Children’s Nutrition and Physical Activity Survey (2007 Children’s Survey) is the most recent nationally representative survey, and its analysis will enable the monitoring of changes in food and nutrient intake among Australian children. The summary results from this survey have been described previously [9]. Findings indicated that boys aged 14–16 years were the subgroup least likely to meet the EAR for zinc, while UL were not reported. The objectives of this paper were to expand on these findings and specifically to (1) describe the dietary zinc intake of children by age/gender groups; (2) identify the major food sources of dietary zinc; and (3) compare the contribution of food groups to dietary zinc intake between 1995 and 2007. 

## 2. Methods

### 2.1. 2007 Children’s Survey

Data for the 2007 Children’s Survey, collected by the Commonwealth Scientific and Industrial Research Organisation (CSIRO) and the University of South Australia (UniSA), were obtained with permission from the Australian Social Sciences Archives [10]. Ethics approval for the study was obtained from the Ethics Committees of the CSIRO and UniSA. In brief, households with children aged 2–16 years were randomly selected using a stratified quota sampling scheme by postcodes. Private dwellings from selected postcodes were recruited to the survey using Random Digital Dialling. Only one child per household was selected for the survey with a response rate of 40% of eligible households [11]. 

Two 24-h recalls were collected; a computer-assisted personal interview (CAPI) followed by a computer-assisted telephone interview (CATI). A three-pass 24-h recall method was used to record all food and beverage intakes on the day prior to each interview from midnight to midnight. A food model booklet was provided to estimate food portion sizes. All interviews were conducted by trained interviewers between 22 February 2007 and 30 August 2007 and were intended to represent different days of the week. Dietary data were collected from the primary care-giver for children aged 2–8 years whereas children aged 9 years and older reported their own food intakes [11]. Supplement use was assessed during the 24-h recall, with dosage information obtained mostly from the Therapeutic Goods Administration (TGA), or alternatively, the supplement label or related website. Dietary and supplement data were translated into nutrient intake data using the survey-specific nutrient database, AUSNUT 2007 [12]. 

### 2.2. 1995 National Nutrition Survey

The 1995 NNS was a systematic sub-sample of those selected for the 1995 National Health Survey (NHS). The sample frame for the NHS was a multistage probability sample of private and non-private dwellings, and for the 1995 NNS, a subsample of households were selected from private dwellings, in which up to three eligible respondents per household could be included in the survey [13]. The response rate for the 1995 NNS was 61.4% of eligible participants. The data (Confidentialised Unit Record Files), and permission to undertake further analysis, were provided by the Australian Bureau of Statistics.

A face-to-face three-pass 24-h dietary recall was used to obtain dietary information, similar to the method used in the 2007 survey but only one day data were collected on all subjects. Respondents were asked to estimate their portion sizes using standardised measuring guides including cups, spoons, ruler, measuring sticks, a grid, different sized shapes and containers, and photos of selected food items. Children aged 12 years and older reported their own food intake data, children aged 5–11 years reported with assistance of a parent while food intake of children aged 2–4 years old was reported by a parent [13]. Data were collected between February 1995 and March 1996.

### 2.3. Under- and Over-Reporters

Misreporters were identified and excluded from both surveys for the comparative analysis. The Goldberg cut-off values were applied to exclude under-reporters and over-reporters, based on the measured (or otherwise estimated) physical activity levels (PAL) and compared with the ratio of reported energy intake (EI) to calculated basal metabolic rate (BMR) [14]. PAL was estimated using a validated use-of-time tool [11]. Recalls with energy intakes outside the cut-off limits (at 95% confidence intervals) were considered implausible and excluded from this secondary analysis. In the 2007 Children’s Survey, 294 (6.1%) out of 4826 children who completed the CAPI were considered under-reporters, 103 (2.1%) over-reporters, and 49 (1.0%) had no weight or height recorded (therefore unable to determine EI: BMR) reducing the sample size to 4380 children. In the 1995 NNS, 113 (4.1%) out of 2682 children aged 2–16 years were considered under-reporters, 87 (3.2%) over-reporters and 47 (1.7%) had no weight or height recorded reducing the sample size to 2435 children.

### 2.4. Statistical Methods

The distribution of dietary zinc intake was determined using two days of 24-h recall data from the 2007 Children’s Survey. Usual intakes were estimated for each individual by adjusting for within-person variability using the Multiple Source Method (MSM) [15]. MSM has been found to produce similar results to the Iowa State University and the National Cancer Institute methods [16]. Usual zinc intakes including and excluding supplementary zinc were considered separately in this analysis. Supplementary zinc was added to dietary zinc intake prior to adjusting for within-person variation. Data were analysed by age and gender groups as determined in the NRV report [7]. The proportion of children meeting their age- and gender-specific EAR and UL were estimated using the EAR cutpoint method [17].

Differences in zinc intakes between the 1995 and 2007 surveys were examined in terms of percentage contribution of major food groups to total zinc intake, excluding supplements. The major food groups and food types analysed were kept consistent between the surveys. Mixed dishes are classified within each major food group and can be directly compared between surveys. For the comparative analysis, only the first day of the 2007 survey (CAPI) was used, to enable more direct comparison with the 1995 survey. Differences between the surveys in food group contributions to total zinc intake were analysed using Fisher’s exact test.

All data were weighted to account for over- or under-sampling to enable representation of the Australian population aged 2–16 years. All analyses were conducted using SPSS version 18.0. *p*-values < 0.05 were considered statistically significant.

## 3. Results

### 3.1. Zinc Intakes; 2007 Children’s Survey

Dietary zinc intakes, including and excluding zinc from dietary supplements, estimated from two 24-h recalls in 4834 children are shown in Table 1 according to age and gender subgroup. For all children combined, the mean zinc intake (excluding supplements) was 10.2 mg per day. Zinc-containing supplements were used by 5.4% of children, with most supplementary zinc consumed being relatively low (median 2.0 mg; range 0.1–129 mg). Few children (0.1%) took a dietary supplement that contained more than 25 mg zinc. Dietary supplements contributed approximately 2% to total zinc intake for all children. The percentage of children who met the EAR and those who exceeded the UL are shown in Table 2. Nearly all children aged 2–13 years (>98%) and girls aged 14–16 years (>92%) met the EAR for zinc. Boys aged 14–16 years were at highest risk of inadequate intakes with over 25% not meeting the EAR for zinc, regardless of supplementary zinc intake. Over three-quarters of children aged 2–3 years exceeded the UL for zinc, compared to approximately 15% of children aged 4–8 years and less than 1% of children aged 9–16 years. The percentage of children exceeding the UL remained high when supplement users were excluded.

**Table 1:** Zinc intake (mg/day) of Australian children by age and gender subgroup; 2007 Children’s Survey *.

					Percentile				
		*n*	Supplements	Mean (SD)	10	25	50	75	90
**Boys** ****									
	**2–3 years**	622	Excluded	8.6 (2.0)	6.3	7.2	8.5	9.8	11.4
			Included	8.8 (2.2)	6.4	7.3	8.6	10.0	11.7
	**4–8 years**	640	Excluded	9.9 (2.3)	7.2	8.4	9.7	11.2	12.7
			Included	10.2 (2.6)	7.3	8.5	9.9	11.4	13.2
	**9–13 years**	579	Excluded	11.2 (2.8)	7.8	9.4	11.0	13.0	15.0
			Included	11.4 (3.0)	7.9	9.5	11.1	13.2	15.2
	**14–16 years**	596	Excluded	13.3 (4.1)	8.9	10.6	12.8	15.2	18.3
			Included	13.7 (4.3)	9.0	10.9	13.1	15.6	18.8
**Girls ** ****									
	**2–3 years**	569	Excluded	8.3 (2.1)	5.8	6.9	8.1	9.4	10.9
			Included	8.6 (2.4)	5.9	7.1	8.4	9.8	11.5
	**4–8 years**	623	Excluded	9.0 (2.1)	6.7	7.5	8.8	10.1	11.9
			Included	9.2 (2.3)	6.8	7.8	8.9	10.3	12.2
	**9–13 years**	640	Excluded	10.0 (2.8)	6.9	8.2	9.6	11.4	13.7
			Included	10.2 (3.0)	6.9	8.3	9.8	11.7	14.0
	**14–16 years**	565	Excluded	9.8 (2.7)	6.4	7.9	9.7	11.3	13.1
			Included	10.3 (3.5)	6.5	8.2	10.0	11.8	13.9
**Total** ****		4834	Excluded	10.2 (3.0)	6.8	8.1	9.7	11.7	14.0
			Included	10.4 (3.3)	6.9	8.3	9.9	12.0	14.4

* using 2-day adjusted data.

**Table 2:** Percentage of children meeting the Estimated Average Requirement (EAR) and exceeding Upper Level of Intakes (UL) for zinc; 2007 Children’s Survey *.

		Supplements	EAR (mg)	% Meeting EAR	UL (mg)	% Exceeding UL
**Boys**						
	**2–3 years **	Excluded	2.5	100	7	79.1
		Included		100		81.0
	**4–8 years**	Excluded	3.0	100	12	15.5
		Included		100		19.4
	**9–13 years**	Excluded	5.0	99.2	25	0
		Included		99.2		0.1
	**14–16 years**	Excluded	11.0	71.2	35	0
		Included		74.3		0
**Girls **						
	**2–3 years**	Excluded	2.5	100	7	74.1
		Included		100		75.8
	**4–8 years**	Excluded	3.0	100	12	9.3
		Included		100		11.6
	**9–13 years**	Excluded	5.0	98.8	25	0
		Included		98.9		0.2
	**14–16 years**	Excluded	6.0	92.3	35	0
		Included		92.9		0

* using 2-day adjusted data.

### 3.2. Contribution of Food Sources to Zinc Intake

In 2007, the three major food groups that contributed most to zinc intake were meat, poultry and game products/dishes (meat and poultry), “milk products/dishes”, and “cereals and cereal products” together accounting for 68% of total zinc intake. Individual food types that at least 5% to total zinc intake were: beef/veal/lamb cuts, milk, breakfast cereals, breads, cheese, and vegetables for 4–8 years old; beef/veal/lamb cuts, milk, breakfast cereals, breads, vegetables, and takeaway burgers/pizza/pasta for 9–13 years old; beef/veal/lamb cuts, breakfast cereals, milk, takeaway burgers/pizza/pasta, breads,vegetables, and beef/veal/lamb mixed dishes for 14–16 years old boys; and beef/veal/lamb cuts, milk, vegetables, breads, cheese,takeaway burgers/pizza/pasta and breakfast cereals for 14–16 years old girls.

**Figure 1:**
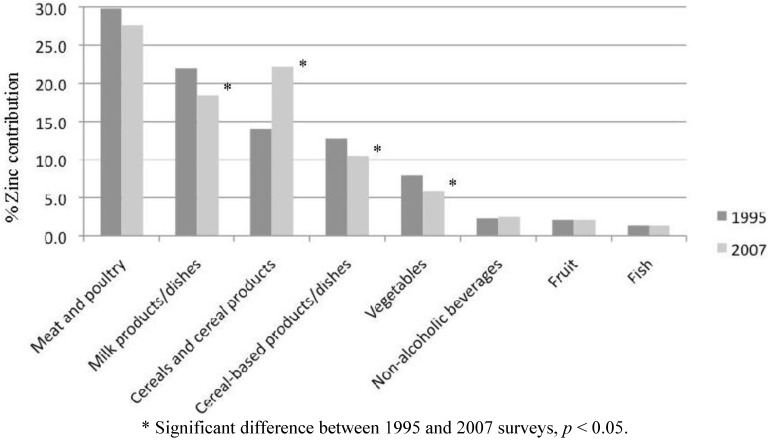
Percent contribution of zinc intake among Australian children aged 2–16 years from major food groups: comparison of 1995 and 2007 surveys.

The major food sources of zinc in the diets of Australian children in 1995 and 2007 are listed in Table 3. Total zinc intakes were higher in 2007 than 1995, due partly to zinc derived from “cereals and cereal products”. The relative contributions of the major food groups to zinc intake are illustrated in Figure 1. The contribution of “cereals and cereal products” has increased significantly since the 1995 survey. Conversely, the contributions of “milk products/dishes”, “cereal-based products/dishes” and “vegetable products/dishes” have decreased over this time period.

**Table 3:** Food sources of zinc (mg/day) by age and gender subgroup of Australian children *; NNS 1995 and 2007 Children’s Survey.

		2–3 years		4–8 years		9–13 years		14–16 years Boys		14–16 years Girls	
		1995	2007	1995	2007	1995	2007	1995	2007	1995	2007
*n*		302	577	842	1506	832	1462	240	437	219	398
**Meat, poultry and game products/dishes**		**1.47**	**1.74**	**2.09**	**2.29**	**3.39**	**3.19**	**4.14**	**5.15**	**2.96**	**2.97**
	Beef, veal, lamb cuts	0.26	0.86	0.44	1.08	1.05	1.47	1.56	2.50	0.94	1.43
	Beef, veal, lamb mixed	0.52	0.25	0.68	0.23	1.03	0.47	0.87	0.86	0.92	0.36
	Pork cuts	0.02	0.04	0.07	0.04	0.19	0.06	0.31	0.11	0.04	0.10
	Pork mixed dishes	0.00	0.01	0.04	0.01	0.02	0.01	0.08	0.01	0.06	0.03
	Poultry cuts	0.12	0.16	0.19	0.22	0.21	0.34	0.38	0.42	0.36	0.38
	Poultry mixed dishes	0.07	0.06	0.15	0.15	0.18	0.21	0.20	0.44	0.22	0.15
	Sausages	0.28	0.17	0.32	0.21	0.45	0.28	0.39	0.29	0.23	0.19
	Ham, bacon	0.09	0.14	0.12	0.23	0.14	0.23	0.26	0.35	0.10	0.23
	Luncheon meat	0.09	0.06	0.08	0.10	0.09	0.09	0.08	0.12	0.03	0.09
	Offal	0.00	0.00	0.00	0.00	0.01	0.02	0.00	0.00	0.06	0.00
**Fish and seafood products/dishes**		**0.06**	**0.08**	**0.15**	**0.18**	**0.13**	**0.14**	**0.13**	**0.13**	**0.15**	**0.12**
**Eggs products/dishes**		**0.06**	**0.07**	**0.08**	**0.09**	**0.09**	**0.08**	**0.14**	**0.11**	**0.07**	**0.10**
**Seed and nut products/dishes**		**0.06**	**0.05**	**0.09**	**0.05**	**0.07**	**0.12**	**0.06**	**0.09**	**0.07**	**0.10**
**Milk products/dishes**		**2.26**	**2.06**	**1.86**	**1.78**	**2.03**	**1.88**	**2.46**	**2.41**	**1.73**	**1.77**
	Milk	1.54	1.21	1.18	0.97	1.25	1.08	1.64	1.43	0.87	0.88
	Cheese	0.39	0.52	0.36	0.54	0.43	0.52	0.49	0.73	0.53	0.65
	Yoghurt, custard	0.21	0.29	0.16	0.20	0.11	0.14	0.08	0.16	0.15	0.16
	Soy milk	0.04	0.07	0.01	0.03	0.01	0.02	0.01	0.04	0.00	0.03
**Cereals and cereal products**		**0.93**	**1.81**	**1.13**	**2.10**	**1.43**	**2.49**	**1.73**	**3.39**	**1.26**	**1.92**
	Breads	0.50	0.63	0.66	0.80	0.80	0.81	0.89	1.10	0.67	0.82
	Breakfast cereals	0.22	0.79	0.26	0.91	0.33	1.05	0.45	1.67	0.24	0.54
	Pasta, noodle	0.04	0.13	0.05	0.16	0.06	0.19	0.05	0.21	0.10	0.21
	Rice	0.08	0.09	0.12	0.10	0.16	0.17	0.25	0.22	0.20	0.17
**Cereal-based products/dishes**		**0.54**	**0.47**	**0.90**	**0.83**	**1.25**	**1.24**	**2.34**	**2.13**	**1.48**	**1.32**
	Meat pie, sausage rolls, *etc.*	0.15	0.09	0.26	0.18	0.29	0.25	0.98	0.51	0.29	0.38
	Takeaway burgers, pizza, pasta	0.23	0.16	0.37	0.37	0.68	0.65	1.07	1.27	0.90	0.60
**Fruit products/dishes**		**0.22**	**0.26**	**0.23**	**0.23**	**0.20**	**0.20**	**0.15**	**0.18**	**0.16**	**0.18**
**Vegetable products/dishes**		**0.42**	**0.40**	**0.54**	**0.47**	**0.88**	**0.68**	**1.11**	**0.94**	**0.88**	**0.83**
**Non-alcoholic beverages**		**0.17**	**0.14**	**0.19**	**0.21**	**0.23**	**0.29**	**0.24**	**0.34**	**0.23**	**0.36**
**TOTAL**		**6.56**	**7.71**	**7.71**	**8.98**	**10.24**	**11.16**	**13.38**	**15.87**	**9.51**	**10.61**

* misreporters excluded.

For children aged 2–3 years, “milk products/dishes” was the main food group contributing to zinc intake in 1995 and 2007, at 34% and 27%, respectively. The second and third highest contributors were “cereals and cereal products” (24%) and “meat and poultry” (23%) in 2007, compared to “meat and poultry” (22%) and “cereals and cereal products” (14%) in 1995. In 2007, the food types that contributed most to zinc intake for 2–3 years old were (in descending order, all contributing at least 5% to total zinc intake): milk; beef/veal/lamb; breakfast cereals; breads; cheese; and vegetables.

For older children (4–16 years), “meat and poultry” was the highest contributor to zinc intake in both 1995 (27%–33%) and 2007 (26%–33%). In 1995, this was followed by “milk products/dishes” (18%–24%); and “cereals and cereal products” (13%–15%) or “cereal-based products/dishes” (12%–17%), while in 2007 “cereals and cereal products” (18%–23%) were the second highest contributors followed by “milk products/dishes” (15%–20%) and “cereal-based products/dishes” (9%–13%). 

## 4. Discussion

The results of the 2007 Children’s Survey show that most children are meeting zinc requirements with the exception of boys aged 14–16 years. Conversely, many children aged 2–3 years are exceeding the upper levels of intake. This is a major change from the 1995 NNS where adolescent girls aged 12–18 years were found to be at highest risk of inadequate zinc intakes [8]. In addition, the comparative analysis examining food sources of zinc between the two surveys shows a change in the contribution of food types to zinc intake. Although the “meats and poultry” food group remains the main contributor to children’s zinc intake, cereals and cereal products now replace milk products as the second highest contributing food group. 

Usual zinc intakes, estimated using two 24-h recalls, of children aged 2–13 years were adequate to meet the age and gender specific EARs. However, in the 14–16 years old age group, the prevalence of boys and girls failing to meet the EAR was 8% and 29%, respectively. The inclusion of dietary supplements did not substantially alter these results. The prevalence of usual zinc intakes below the EAR is the recommended *dietary* indicator to identify population groups at elevated risk of zinc deficiency [18]. As the NRVs for zinc were revised in 2005, it is important to re-evaluate available data on zinc intake. The revised average requirements were increased for adolescent boys from 8.4 mg/day (calculated as 70% of RDI) to 11 mg/day (EAR) and decreased for adolescent girls from 8.4 mg/day (calculated as 70% of RDI) to 6 mg/day (EAR). If these values (*i.e.*, 70% RDI) were applied to the 2007 Children’s Survey, the proportion of 14–16 years old boys and girls failing to meet these requirements would be 7% and 30%, respectively. These estimates are the reverse of our current findings. Uncertainty remains in setting the EAR values for toddlers, children and adolescents. These EARs are currently based on those of adults, adjusting for reference body weights, with an additional requirement for growth using absorption factors of 24% for boys and 31% for girls [7]. 

In contrast to the 2007 Children’s Survey **results,** adolescent boys (aged 12–18 years) were not at risk of inadequate zinc intakes in the 1995 NNS [8]. Instead, adolescent girls were at highest risk with 43% of 12–15 years old and 39% of 16–18 years old not meeting 0.7 RDI (8.4 mg). A similar finding was reported in the Canadian Community Health Survey undertaken in 2004 with approximately 20% of adolescent girls not meeting the EAR [19], however, the Canadian EAR are different to the Australian EAR.

Other dietary surveys in Australia have identified low zinc intakes in pre-school children and adolescents [20,21] but biochemical screening of pre-school children across a range of socioeconomic status showed that plasma zinc concentrations were within the normal range [22]. In the New Zealand Children’s National Nutrition Survey, the mean zinc intakes for children of European origin (*i.e.*, excluding those of Pacific and Māori origin) were similar to those observed in the present survey [23]. The overall prevalence of inadequate zinc intakes in this New Zealand survey was low although not all sub-groups were analysed. Furthermore, direct comparisons of our findings with the US NHANES III results were not possible because of differences in the calculated average requirements that were used to assess the adequacy of zinc intake [24].

The UL is the highest average level of daily intake that is likely to pose no risk of adverse health effects and as intake increases above the UL, the potential risk of adverse effects increases. A high percentage of 2–3 years old children exceeded the UL of zinc intake of 7 mg. Children in older age groups were less likely to exceed the UL. In adults, the ingestion of zinc supplements beyond the UL results in nausea and gastric distress as well as a decrease in plasma HDL-cholesterol concentrations in normolipidaemic individuals [25,26]. The main adverse effect, however, is due to the antagonistic relationship between zinc supplementation and its impact on copper absorption, resulting in a decrease in copper-related functions including the activity of superoxide dismutase [27].

In children the derivation of the UL is controversial. The UL for infants is based on the study of Walravens and Hambidge [28] where the No Observed Adverse Effect Level (NOAEL) was set at 4 mg per day and adjusted for older children based on relative body weight [17]. The UL applies to total zinc intake from food (including fortified foods) and supplements. The findings in the present analysis, that over 79% of children aged 2–3 years are exceeding the UL, are difficult to interpret in the absence of biomarker evidence. In the US Continuing Survey of Food Intakes by Individuals (CSFII), 51% of preschool children (age 1–3 years) exceeded the zinc UL. This percentage decreased substantially (3%) among older children (age 4–5 years) [29]. It has been shown by Gibson *et al.* [5] and Ruz *et al.* [30] that an intake of zinc (including supplements) of 16.7 mg/day and 17.1 mg/day, respectively, in children 8 years and under had no impact on biomarkers of copper status. Thus in the absence of any reported adverse effects, the findings from the 2007 survey of Australian children and the American CSFII support the suggestion that the current UL for the 2–3 years age group is underestimated and should be reviewed [31].

The use of supplementary zinc was relatively low among children and the majority of supplements provided only small amounts of zinc to the diet. Few children consumed large amounts of supplementary zinc far exceeding the UL. However, the frequency of such supplemental intakes cannot be established from the current data. Further investigation of zinc supplementation practices among children is warranted.

Biochemical and functional indicators were not available in the 2007 Survey thus limiting a full assessment of zinc status in this population. In the absence of any biomarker data, there is insufficient evidence to determine whether the observed low zinc intake in boys age 14–16 years or intakes above the UL for children 2–3 years old is reflective of abnormal zinc status.

The current major food sources of zinc in the diets of Australian children include meat and poultry, cereals and cereal products, and milk products. The contribution of cereals and cereal products to total zinc intake has increased significantly between the 1995 and the 2007 surveys. This is due to an absolute increase in zinc consumption from these food groups, as zinc intakes from meats and poultry as well as milk products/dishes remained relatively stable. Zinc fortification of breakfast cereals has become more widespread since its introduction in the early 1990s. The AUSNUT99 database (used in the 1995 NNS) listed only six zinc fortified breakfast cereals (out of a possible 64), compared to 33 (out of a possible 94) in the AUSNUT2007 database. Zinc fortified breakfast cereals contain approximately 6 mg zinc/100 g while their unfortified counterparts contain approximately 1 mg/100 g. 

The identification of foods types that contribute most to zinc intake may be helpful in the planning and assessment of children’s diets. The three foods that contributed most to zinc intake for all age groups included beef/veal/lamb cuts, milk, and breakfast cereals. Milk was a higher contributor in the diets of 2–3 years old while beef/veal/lamb cuts were the highest contributors in all other age groups. In addition, the contribution of zinc from milk is underestimated as some milk items are classified under the non-alcoholic beverage food group as milk in tea, coffee or with beverage flavourings. 

Although zinc is found widely in the food supply, its bioavailability from different foods is highly variable. Rich sources of zinc include foods from animal sources such oysters/seafood, meat and dairy products. These sources have made a similar or smaller contribution in the 2007 survey compared to the 1995 NNS. In contrast, the contribution of cereals-based foods has increased since 1995. Cereals are rich in phytate, which binds zinc in the intestine and reduces its absorption [32]. The early cases of zinc deficiency were associated with high phytate-containing foods: unleavened bread from unrefined wheat flour as a dietary staple, and beans [2]. The molar ratio of phytate: zinc in the diet has been proposed as a predictor of zinc bioavailability and ratios greater than 15 have been associated with suboptimal zinc status [33]. In a recent dietary survey of adults, we reported that the intake of phytate was positively related to the intake of dietary fibre [34]. In view of the substantial and increased contribution of cereals in 2007 as compared to 1995, further research is needed to determine the bioavailability of zinc from fortified fibre-rich breakfast cereals. In addition, the phytate content of the Australian food supply, particularly the higher inositol phosphates (IP-6 and IP-5) responsible for binding zinc [35], requires analysis.

A number of limitations must be acknowledged. Firstly, the dietary data collected for the 2007 Children’s Survey consisted of two 24-h recalls, and although adjusted for within-person variation, may not be representative of “usual” intake, particularly for supplementary zinc intake [36]. The adjusted zinc intakes described in this study are lower than those reported in the Main Findings report of the 2007 Children’s Survey [9]. This may be due to the dietary intakes of zinc being lower in the second 24-h recall (CATI) compared to the first day (CAPI), and to the different method used to estimate “usual” intake. In addition, some methodological differences exist between the 1995 and 2007 surveys, including differences in survey time frame, sampling frames, children self reporting their dietary intake at a younger age in the 2007 Children’s Survey and different tools used in the estimation of portion sizes. The food composition databases used were those specific to the national surveys analysed. The analytical method of estimating zinc content in foods has changed from dry-ashing and flame atomic absorption spectrophotometry (AAS) during 1980s to 1990s to current methods of inductively-coupled plasma analysis, which has little effect on analysed zinc values (personal communication, FSANZ, 2012). Only 1 day of dietary intake was available for the comparative analysis of food sources contributing to zinc intake but this is sufficient to provide an estimate of usual average intake of groups [37]. Strengths of the study included nationally representative data based on age, gender and region, the use of similar dietary methodologies and methods of analysis.

## 5. Conclusions

The 2007 Children’s Survey identified boys aged 14–16 years as being at risk of insufficient intake while children 2–3 years consumed zinc in amounts beyond the UL. In addition, absolute intakes of zinc had increased since the previous national survey, largely due to an increased zinc intake from cereals and cereal products due to fortification. Further information such as biomarkers of nutritional status and anthropometric characteristics is required to confirm the zinc status of these groups.
